# Structural field margin characteristics affect the functional traits of herbaceous vegetation

**DOI:** 10.1371/journal.pone.0238916

**Published:** 2020-09-17

**Authors:** Cian Blaix, Anna-Camilla Moonen

**Affiliations:** Group of Agroecology, Institute of Life Sciences, Scuola Superiore Sant'Anna, Pisa, Italy; Ghent University, BELGIUM

## Abstract

**Background:**

Field margins are ecologically important to an agroecosystem as they are a source of biodiversity. They can be composed of a diverse flora which may offer resources to a wide range of insects and birds. The vegetation composition of field margins is determined by soil characteristics, management, and landscape structures. However, little is known about the effect of individual field margin components such as ditches, grass strips, shrubs and trees, and the overall margin’s complexity, on the vegetation composition and its functional effect and response traits.

**Methods:**

This paper reports on the effects of field margin component typology (ditches, grass strips, shrubs, trees, and vehicle tracks) and complexity (the number of components), on the herbaceous vegetation of field margins. Forty field margins were sampled in 2016 in a 200 ha. organic mixed arable livestock farm.

**Results:**

The factor which was identified as having the most effect on vegetation composition was adjacent land-use type, which reflected the margins’ management regime. However, field margin components were found to affect vegetation response and effect traits. Tree components had less grassweeds than vehicle tracks while more complex field margins also had less grassweeds than simple field margins near cropped fields, most likely due to the lower availability in light and less disturbance from vehicles. Simple grassy margins produced a high proportion of hymenoptera flowers.

**Discussion:**

These results highlight the importance of field margin components in maintaining a high diversity of vegetation typologies differing in effect traits that are relevant for the provisioning of ecosystem services, such as supporting pollen and nectar requirements of beneficial insects, as well as their importance in determining the presence of weed species that could potentially invade the cropped fields.

## Introduction

Semi-natural habitats are considered as key ecological features in agroecosystems. They may offer resources to beneficial arthropods as well as being important for biodiversity conservation [1–3]. Field margins are one of the most common type of semi-natural habitat in agroecosystems. These linear features may consist of components which differ depending on the physical characteristics of the vegetation (e.g. shrubs, trees and grass strips) or other physical structures (e.g. ditches, stone walls and raised banks). The variety of components that can be found in field margins means that a field margin may be very simple, for example, composed of a single homogeneous grass strip, or complex with many different component types in the same margin. The presence or absence of certain component types in a field margin may influence the vegetation composition of the margin due to their effect on the intensity of different stress types (e.g. water, light, or temperature). Indeed, stress is known to be an important environmental gradient in structuring plant communities where the tolerance of species to a certain stress will determine if it can inhabit a selected habitat [4]. Furthermore, the accumulation of field margin component types in an individual margin may lead to a greater diversity of environmental conditions which are suitable to a variety of species. An analysis of CSR strategies used by species can help identifying ecological filters that structure vegetation compositions [5]. These strategies describe the how species adapt to their environment [4].

Field margin composition may also directly affect services provided by arthropods. For example, a line of trees in field margins can act as a windbreak and favour the presence of flying pest predators and pollinators [6]. Field margins may also impact neighbouring species communities as they can act as both barriers and corridors to the movement of arthropods towards cultivated fields and insect-pollinated flora [7, 8]). Besides the nature conservation functions attributed to all semi-natural habitats and their ecological role in agroecosystems, field margins have other practical agronomical functions such as livestock confinement, regulation of access to fields and supporting beneficial insects for pest control and crop pollination [7]. Due to the fact that they are ecotones, they may host a high plant species diversity and, as a result of different physical characteristics, the vegetation composition may be highly diversified [5, 7]. They may offer a transitional habitat from a more natural habitat, such as a forest, to fields which are more disturbed. This leads to a gradient with a less acute change in disturbance and may also lead to a gradient in stress (e.g. more humid forest to drier fields). This can impact habitat quality which plays an important role in hosting beneficial insects [9, 10]. The analysis of the ecological and biological functions of biodiversity is needed in order to guide decisions on agroecological habitat and land use management. Agroecological land use management aims to increase the magnitude of agroecosystem services provided by the naturally occurring biodiversity. Therefore, the most functional biota in the agroecosystem need to be identified and managed and this can be done through a functional trait analysis of the present species in relation to the objectives posed for the studied agroecosystem.

Different semi-natural habitats and, in particular, field margins can have different species compositions, but their functions can be similar. By classifying organisms based on their ecological characteristics or functional traits [11], it is possible to analyse ecosystem services and trade-offs independently of species composition. These traits can be divided into two types; response traits and effect traits (see Fig 1 in [11]). The response traits refer to the way plants respond to certain environmental factors while the effect traits are the contributions of plants to ecosystem functioning. This framework can be used to identify traits that are associated with ecosystem services and predict changes in service delivery in response to changes in functional traits [12]. Storkey et al. [13] used this framework to find that the presence of ruderal plants increased the abundance of invertebrate food for farmland birds while the framework has also been used to choose the most effective cover crop composition for nutrient provisioning [14]. The use of functional traits has a lot of potential in agroecology [15–17]. For example, by identifying the response traits associated with plants that are competitive with crops, it is possible to identify the factors that affect their abundance in fields and adopt field management strategies accordingly. Functional traits can also be used to identify the potential services that plants can provide to agroecosystems [18]. Compiling information on the abundance of vegetation based on the type of flower they produce and on flowering period allows for an estimation of the potential a biotope has of attracting certain type of beneficial insects at a certain period of the year. This information is useful to harmonize the timing of a service with the cropping cycle. Although studies have looked at the effect of management and field margin component type on vegetation composition and life history traits in field margins (e.g. [19–24]), there is a lack of knowledge on the effect of field margin component type on plant communities and their functional traits.

The aim of this study is to analyse the effect of structural field margin characteristics, particularly focusing on individual field margin components, on the species composition in the herbaceous part of the field margins, while taking into account soil characteristics and vegetation management. Since complex landscape structures can impact the vegetation compositions of field margins, we decided to conduct the study at the farm scale to allow for the observation of the effects of field margin characteristics [20]. We hypothesize that structural field margin characteristics influenced field margin vegetation composition of the herbaceous layer. We also tested if these factors were associated with particular plant response and effect traits affecting the abundance of potential crop weeds and the provisioning of floral resources for beneficial insects. The final objective of this approach is to show how field margin components are related to plant community functional traits, allowing researchers and land managers to estimate potential ecosystem services that can be provided by field margins based on easy to assess visual characteristics.

## Materials & methods

The study was conducted in an organic mixed livestock farm of circa 200 ha. of arable crops, pastures and forage crops in the regional park of Migliarino, San Rossore and Massaciuccoli in the province of Pisa, Italy (S1 Fig). Field permit for sampling was granted by Parco Regionale Migliarino—San Rossore—Massaciuccoli (permit number: ns. prot. 6152/7-2.1). The sampling site is located near the southern confinement of the park, 5 km west of Pisa (43°41’N, 10°19’E). It is surrounded by natural woodland on the North, West, and partly on the East side, and it is bordered by a poplar plantation and the river Arno on the South side. The northern part of the study area is dominated by arable crops and meadows set aside for hay, while the southern part is characterised by pastures. The soil type is mostly loam and sandy loam. A summary of the soil variables measured is displayed in S1 Table.

### Field margin

Forty herbaceous field margin strips were chosen for the study. They were selected to include eight replicates of the five different field margin component types: 1) no additional structure next to the herbaceous strip, 2) a ditch, 3) a herbaceous vehicle track, 4) shrubs and 5) trees (see S2 Table for a detailed definition of the component types and S2–S4 Figs for photographic examples). Only field margins of at least 60 m in length and 1 m wide were considered. Any homogeneous vegetation strip wider than 20 m adjacent to a field margin was considered as a land-use type. Field margins were classified into four categories based on their component composition: field margins with grassy components only (containing only grass strips and/or vehicle tracks); field margins with a grassy component and a woody component (shrubs and/or trees); field margins with a grassy component and a ditch; field margins with a grassy component, a woody component, and a ditch. The complexity of the field margins was calculated, which consisted of the number of component types in each field margin. The adjacent land use was used as a proxy for field margin management because the two variables were very closely related. Since the farm is part of a natural park, the area where arable crops are grown is fenced to exclude wild animals. Margins are mowed twice a year (in spring and in autumn) when fields are accessible. The remaining area consists of permanent grassland used by horses and field margins can be grazed and are not mowed as frequently. The last category of land use is white roads. Margins are mowed twice a year but the vegetation in these margins is disturbed by dust, especially in the dry summer period. Therefore, in the selected study area, field margin management was closely correlated to adjacent land use and only land use was included in the analyses.

### Sampling

Sampling was done in the herbaceous part of the component. Vegetation surveys were carried out in June 2016 in 1-m wide transects of 25 m in length. The sampling points were at least 80 m apart and located as close to the middle of the field margin as possible. Plant species abundance was expressed in percentage cover. Only species with >1% cover were sampled since in trait-based approaches the most abundant species determine the main traits expressed by the vegetation [25]. In each transect, three soil samples were taken with a soil probe of 3.5 cm in diameter, up to 10 cm depth, and bulked. They were stored in plastic bags and brought to a laboratory in cool boxes for analysis. They were used to determine soil nitrogen (N) content as well as soil organic matter (SOM), granulometry, and water-holding capacity.

### Species traits and functional groups

Functional groups were formed for analysis based on species traits. Plants were grouped in terms of typology considering their weediness, whether they are monocotyledons or dicotyledons, and their physical structure. Five plant species types were identified: ‘dicot weeds’ (dicotyledon weeds), ‘grassweeds’ (monocotyledon weeds), ‘dicots’ (non-weed dicotyledons), ‘grasses’ (non-weed monocotyledons), and ‘woody’ species. ‘Woody’ species are the seedlings or stolons of trees or shrubs. Species were classified as ‘weeds’ or ‘grassweeds’ if they were encountered inside the cropped fields in the study area and by using expert knowledge. The database constructed by Pierce et al. [26] was used to determine the CSR ratio of the plant species. The CSR ratio quantifies the competitive ability, the stress tolerance, and the tolerance to disturbances of a species based on Grime’s CSR strategies [4].

Effect traits were acquired using the TR8 package in the R software (3.3.1) [27,28]. The package retrieves data on plant traits from a range of databases. The following functional effect groups were formed to estimate the capacity of the vegetation to support beneficial insects: Müller’s flower class, flower colour and flowering period [29–31]. This information was collected only for insect pollinated species. Müller’s flower class is a classification based on the type of pollination a flower undergoes as well as the accessibility of the reward provided to pollinators [32]. The data on flower class was adapted to form six classes; ‘open nectar flowers’, ‘hidden nectar flowers’, ‘diptera flowers’, ‘lepidoptera flowers’, ‘hymenoptera flowers’, and ‘wind flowers’. Only two species classified as having lepidoptera flowers were recorded and were left out for the analyses of this trait. See S3 Table for a more detailed description of Müller’s flower class. Flowering period indicates during which months the species is in flower in Italy. The cover of species that flower in May, June and/or August were investigated as those species are at or close to full vegetation development during the sampling date. The number of species with a percentage cover above 1% that were in flower in each month was considered to estimate the resilience of the vegetation assemblages in providing floral resources for each month. Flower colour was also considered because it can be related to flower preferences of beneficial insects [33–35]. The data were adapted into four levels; ‘blue’, ‘pink’, ‘white’, and ‘yellow’. The blue category also includes purple flowers while the pink category also includes red flowers as distinction among those sets of colours was difficult and arbitrary.

Regarding species for which no information on a trait was available, the median of the values for species of the same genera was used for quantitative variables and the most common factor level in the genera for qualitative variables. Data on CSR ratios were still missing for five species even after this procedure and, instead, the data were estimated using the regularized iterative FAMD algorithm which consists of a principal component method for imputing missing data [36]. If the median value for species of the same genera as the target species was not available for the Pignatti values, values were obtained or estimated from the database for species of Central and Southern Italy [37].

### Data analysis

A Principal Coordinate analysis (PCoA) was used to differentiate the samples based on their vegetation composition using the Bray-Curtis dissimilarity index and a Lingoes correction [38]. The influence of categorical variables, namely, adjacent land-use type, field margin component type, and field margin component composition, on PCoA scores was tested using a distance-based redundancy analysis (dbRDA), a method which combines regression analysis with a PCoA [38]. Due to the hierarchical relationships between adjacent land-use type and component type, and adjacent land-use type and field margin component composition, one dbRDA was performed with all three variables included, to test the effect of adjacent land use type on the PCoA, while two others were executed with the effect of adjacent land-use type partialled out; one dbRDA containing the variable component type, and another containing component composition. Component type and field margin component composition were analysed separately due to the non-independence between variables. Statistical significance of each variable on the ordination was tested using permutation tests. As the permutation test is sequential and the order of the variables affects the test statistics, the reported results for a variable is from the model run with that variable included as the first term in the sequence. For continuous variables, permutation tests were applied to determine the linear relationship between the variables and the first two axes of the PCoA. Variables that were not correlated to any axes were then tested for a non-linear relationship with the two axes using generalised additive models (GAM). Only variables that had a significant association with the axes were plotted in the PCoA biplot. Due to a strong correlation between N and SOM (*r* = 0.91, d.f. = 37, *p* < 0.001), only SOM was considered in the analyses. Factors which had a significant effect in the dbRDA were analysed using species indicator values to determine which species were associated with which factor levels [39]. This analysis calculates an index from 0 to 1 describing how representative each species is to a factor level based on its specificity (how faithful a species is to a group) and its fidelity (how evenly a species is distributed in a group).

Multivariate response models were used in a Bayesian framework to determine the relationship between three field margin characteristics; component type, field margin component composition, and field margin complexity and the functional groups. Due to the non-independent nature of the explanatory variables, they were fitted in separate models. The models also contained adjacent land-use type as group effects. The group effects were modelled as being correlated [40]. Both varying intercept and varying slope models were considered. Selection between the two types was made based on the widely applicable information criterion (WAIC) and leave-one-out cross-validation (LOO-CV) [41]. For most models, response variables consisted of proportions and a Dirichlet distribution was used. If the proportions for each field margin did not sum to one, they were normalized by dividing by the sum. A Poisson distribution was used for the models containing species richness and a beta distribution for the vegetation cover of flowering species. The community weighted means of the proportion of each CSR strategy type were used. Weakly informative priors were applied. Description of the models retained for analyses are available as supplementary information (S4 Table). Posterior means and credible intervals of 89% were used for reporting the results of the models following the example of McElreath [42]. Differences in and between the predicted variables are considered to exist between factor levels when the credible intervals do not overlap. The effect of complexity in field margins adjacent to the road was not plotted as these margins all had the same number of field margin component types. Additional analyses were performed using Pignatti values for light for the interpretation of the results and are included in the supplementary information [30]. These consist of ecological indices equivalent to Ellenberg indicator values [43].

All analyses were performed on R [28]. Ordination analyses were executed using the vegan package [44]. Bayesian models were run using the brms package [45] which provides an R interface for Stan, a statistical modelling platform [46]. The following packages were also used: “DirichletReg” [47], “FD” [48], “ggplot2” [49], “missMDA” [50], “TR8” [27].

## Results

### Variability in vegetation composition

**Fig 1:**
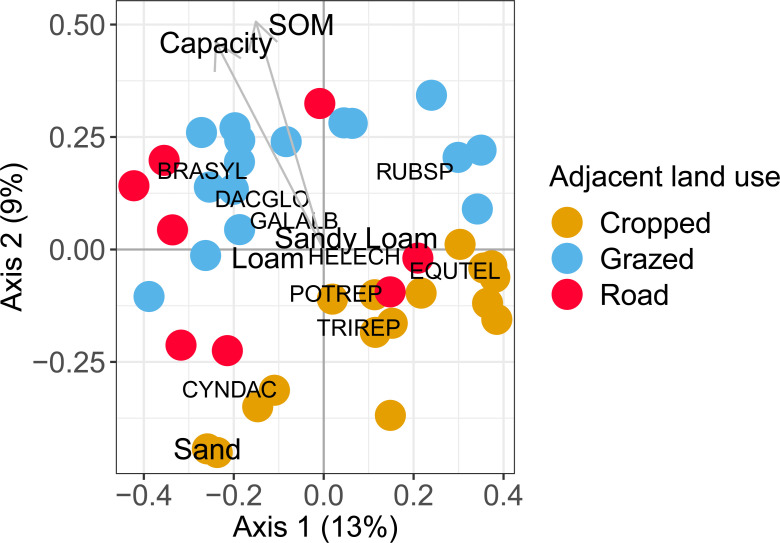
PCoA biplot including the sampled field margins and explanatory variables. Biplot of the PCoA site coordinates fitted with environmental variables and the nine most frequently occurring species. Capacity: field capacity; SOM: soil organic matter. BRASYL: *Brachypodium sylvaticum*; CYNDAC: *Cynodon dactylon*; DACGLO: *Dactylis glomerata*; EQUTEL: *Equisetum telmateia*; GALALB: *Galium album*; HELECH: *Helminthotheca echioides*; POTREP: *Potentilla reptans*; RUBSP: *Rubus* sp.; TRIREP: *Trifolium repens*.

**Fig 2:**
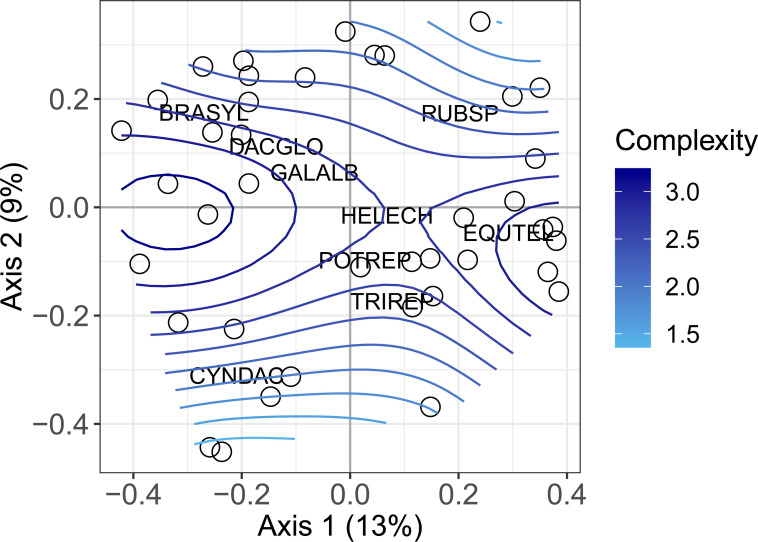
PCoA biplot including the effect of field margin complexity. Biplot of the PCoA site coordinates fitted with GAM smooth function for field margin complexity. BRASYL: *Brachypodium sylvaticum*; CYNDAC: *Cynodon dactylon*; DACGLO: *Dactylis glomerata*; EQUTEL: *Equisetum telmateia*; GALALB: *Galium album*; HELECH: *Helminthotheca echioides*; POTREP: *Potentilla reptans*; RUBSP: *Rubus* sp.; TRIREP: *Trifolium repens*.

A total of 77 species with a cover > 1% were sampled (S5 Table). The most frequently sampled species was *Equisetum telmateia* which was present in 58% of the sampled field margins. Adjacent land-use type significantly affected the plant species composition (*F* = 2.67, d.f. = 2, *p* < 0.001), however, the dbRDA was not significant for component type (*F =* 1.23, d.f. = 4, *p* = 0.05) or field margin component composition (*F =* 1.13, d.f. = 3, *p* = 0.199). The species most associated with field margins adjacent to cropped fields was *Trifolium repens* (indicator value = 0.66, *p* < 0.001) while *Rubus* sp. (indicator value = 0.56, *p* = 0.01) and *Dactylis glomerata* (indicator value = 0.55, *p* = 0.002) were the species most associated with field margins adjacent to grazed field and the road respectively (Table 1). The first two axes of the PCoA explained 22% (1^st^ axis = 13% and 2^nd^ axis = 9%) of the variation (Figs 1 and 2). The main effect of adjacent land-use type on the ordination is seen on the 2^nd^ axis which clearly differentiates field margins adjacent to cropped fields from field margins adjacent to grazed fields (Fig 1). Also, both soil organic matter (*R*^2^ = 0.22, d.f. = 38, *p* = 0.007) and field capacity (*R*^2^ = 0.27, d.f. = 38, *p* < 0.01) increased from the bottom of the biplot to the top. There is also an effect of adjacent land-use type that can be seen on the 1^st^ axis with many margins adjacent to the road and margins adjacent to the grazed fields located on the left side of the biplot and most margins adjacent to cropped fields found on the right. Complexity of the field margins had a significant non-linear effect on the vegetation ordination (adj. *R*^2^ = 0.53, d.f. = 38, *p* < 0.001). Complex field margins were gathered around the centre of the 2^nd^ axis and simple margins were found near the extremities of the 2^nd^ axis (Fig 2).

**Table 1:** Species associated with adjacent land-use type.

Species	Cropped fields		Grazed fields		Road	
	Indval	*p*-value	Indval	*p*-value	Indval	*p*-value
*Trifolium repens*	0.66	<0.001				
*Equisetum telmateia*	0.56	0.007				
*Rubus* sp.			0.56	0.01		
*Dactylis glomerata*					0.55	0.002
*Pulicaria dysenterica*					0.54	0.005
*Brachypodium pinnatum*					0.37	0.015
*Teucrium scorolium*					0.36	0.005
*Hordeum murinum*					0.25	0.034
*Valeriana officinalis*					0.25	0.035

Species with their associated indicator value and *p*-value for each adjacent land-use type. Indval = indicator value.

### Response traits

**Fig 3:**
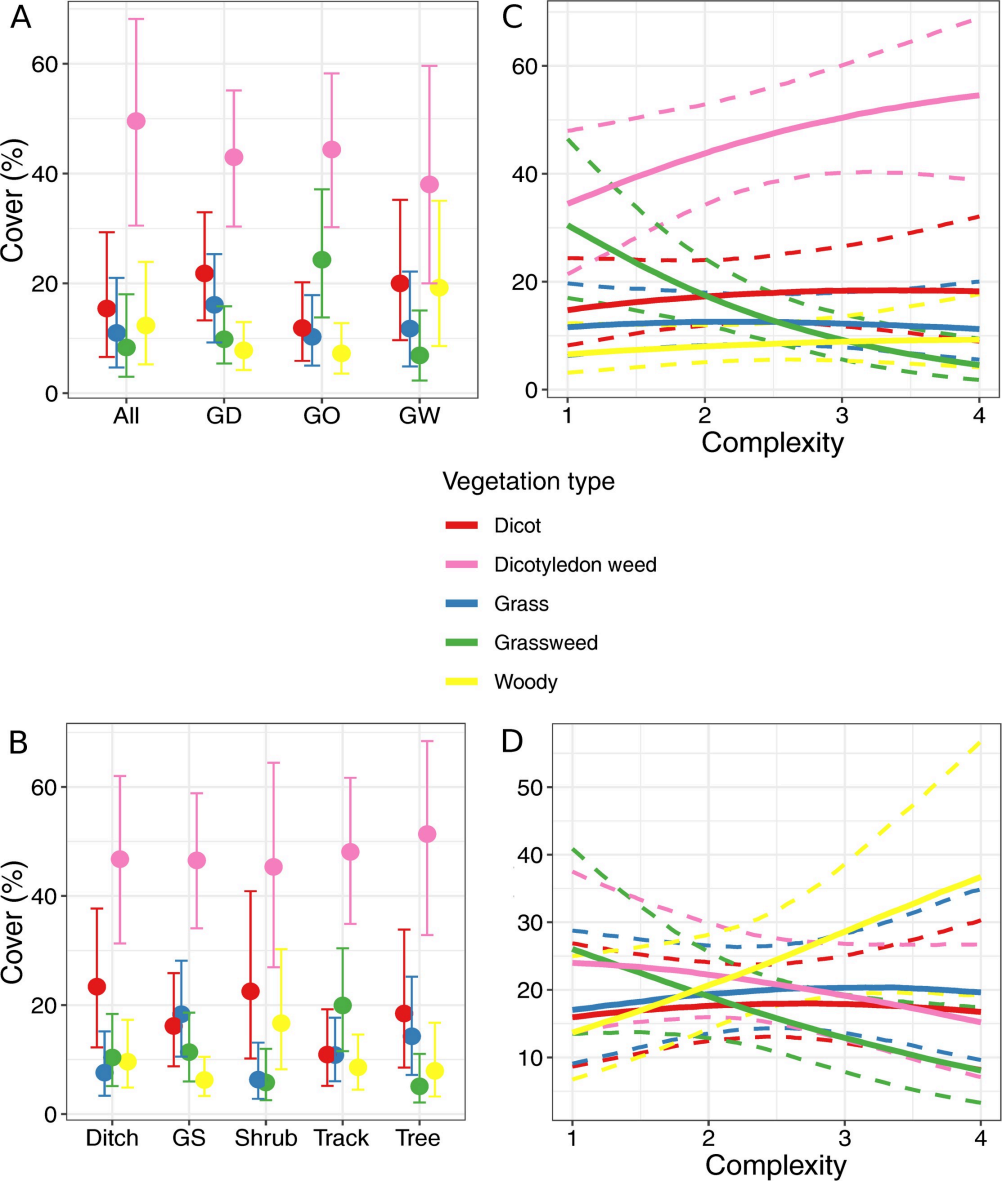
Effect of field margin characteristics on vegetation type. Marginal effects of field margin variables on vegetation type. Error bars and bands represent 89% credible intervals. A: field margin composition; B: field margin component type; C: field margin complexity in cropped fields; D: field margin complexity in grazed fields. GS: grass strip; All: margin contains grassy, woody and ditch components; GD: margin contains grassy and ditch components; GO: margin contains grassy component(s); GW: margin contains grassy and woody components.

Dicotyledon weeds were the dominant vegetation type in the herbaceous part of the field margins (Fig 3A and 3B). Their abundance changed little between field margin composition or component type. However, composition and component type did have an effect on relative abundances of vegetation types. The percentage of dicot weeds in field margins containing ditches, woody and grassy components differed from the percentage of other vegetation type present (Fig 3A). In all other types of field margin composition, the credible intervals (CIs) for dicot weeds overlap with at least one other CI. The grassweed cover was higher than the cover of woody species in field margins composed of an herbaceous margin only. More grassweeds were found in the track component than in the tree component while, in the grass strip, track and tree components, a higher percentage of dicot weed cover was present than dicots (Fig 3B). Complexity influenced grassweeds in field margins adjacent to cropped fields (Fig 3C). Their percentage cover decreased in these field margins with an increase in complexity. Furthermore, an increase in the percentage of cover of dicot weeds with an increase in complexity resulted in more dicot weeds being found than grassweeds in complex field margins. A higher percentage cover of woody vegetation than grassweeds was found in complex margins adjacent to grazed fields (Fig 3D).

**Fig 4:**
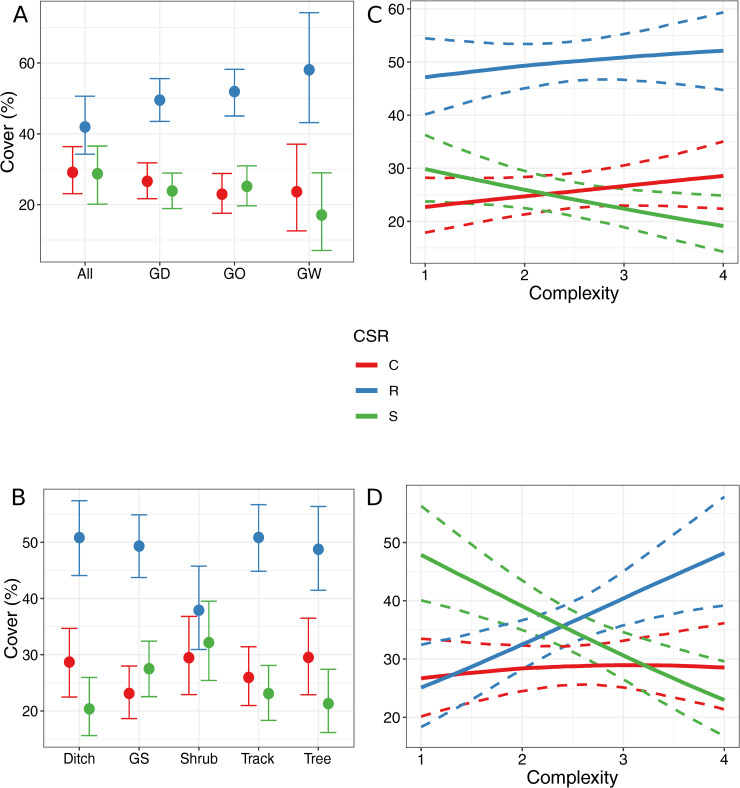
Effect of field margin characteristics on CSR strategy. Marginal effects of field margin variables on Grime’s CSR strategy. Error bars and bands represent 89% credible intervals. A: field margin composition; B: field margin component type; C: field margin complexity in cropped fields; D: field margin complexity in grazed fields. GS: grass strip; All: margin contains grassy, woody and ditch components; GD: margin contains grassy and ditch components; GO: margin contains grassy component(s); GW: margin contains grassy and woody components.

In general, more ruderal species were present in the field margins than stress-tolerant species or competitive species (Fig 4A and 4B). Indeed, CIs for ruderal species did not overlap with CIs for other strategy types except when all of grassy, woody, and ditch components were present (Fig 4A). Similarly, ruderal species were dominant in all component types except for the shrub component (Fig 4B). The abundance of ruderal species increased with field margin complexity at the expense of stress-tolerant species in both cropped and grazed fields (Fig 4C and 4D).

### Effect traits

**Fig 5:**
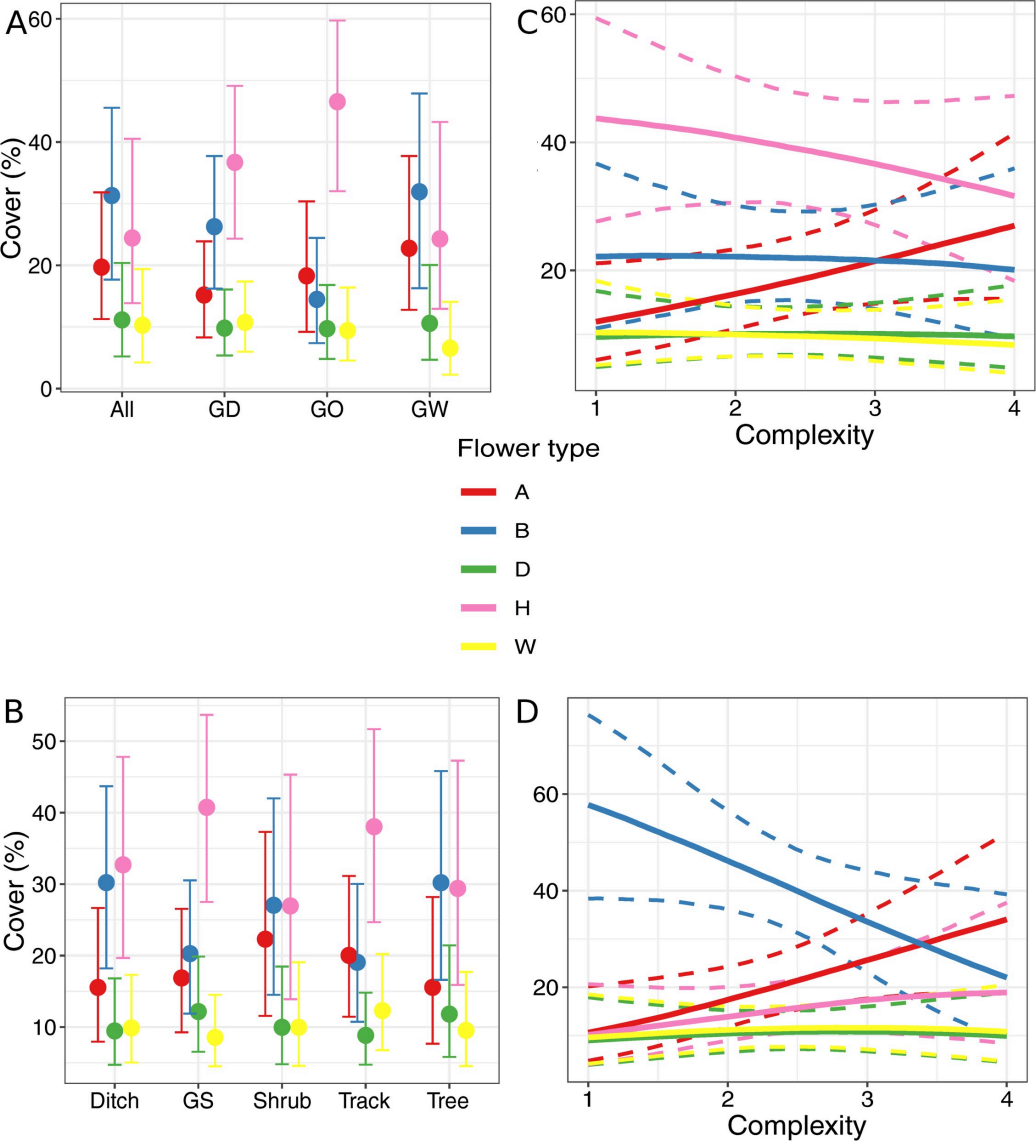
Effect of field margin characteristics on flower type. Marginal effects of field margin variables on flower type. Error bars and bands represent 89% credible intervals. A: open nectar flowers; B: hidden nectar flowers; D: diptera flowers; H: hymenoptera flowers; W: wind pollinated flowers. A: field margin composition; B: field margin component type; C: field margin complexity in cropped fields; D: field margin complexity in grazed fields. GS: grass strip; All: margin contains grassy, woody and ditch components; GD: margin contains grassy and ditch components; GO: margin contains grassy component(s); GW: margin contains grassy and woody components.

Margins with only grassy components had more hymenoptera flowers than any other types of flowers while in other composition types, the CI for hymenoptera flowers overlaps with CIs of at least one other flower type (Fig 5A). More vegetation with hidden nectar flowers was found than vegetation with diptera flowers in margins composed of a ditch and grassy components. Also, a higher percentage of plants with hidden nectar flowers was found than with wind pollinated flowers in margins composed of woody and grassy components. No differences in flower types were detected in shrub and tree components (Fig 5B). In ditch and track components, more plants with hymenoptera flowers were found than plants with wind pollinated flowers or diptera flowers, while a higher percentage cover of vegetation with hymenoptera flowers was found than any other flower type except for hidden nectar flowers in grass strips. In field margins adjacent to cropped fields, the percentage cover of open flowers increases while the cover of hymenoptera flowers slightly decreases with increasing field margin complexity (Fig 5C). Field margins adjacent to grazed fields contained more vegetation with hidden nectar flowers than any other type of flowers in simple field margins, while no difference between flower type percentage cover was found in complex margins (Fig 5D).

**Fig 6:**
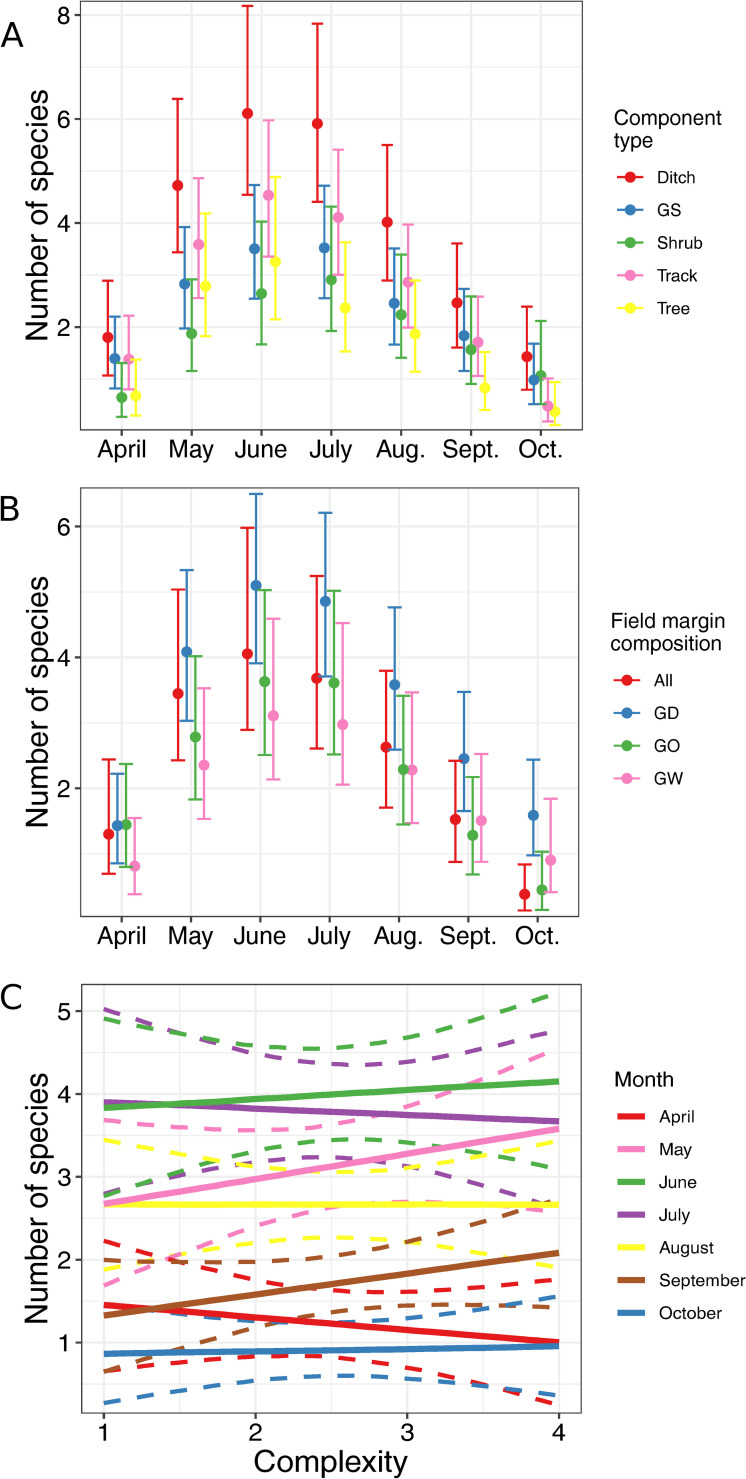
Effect of field margin characteristics on flowering period. Marginal effects of field margin variables on the number of species sampled that flower during each month from April to October. Error bars and bands represent 89% credible intervals. A: field margin composition; B: field margin component type; C: field margin complexity in cropped fields; D: field margin complexity in grazed fields. GS: grass strip; All: margin contains grassy, woody and ditch components; GD: margin contains grassy and ditch components; GO: margin contains grassy component(s); GW: margin contains grassy and woody components; Aug.: August; Sept.: September; Oct.: October.

Field margin component type and field margin composition did not have an influence on the percentage cover of species that are in flower during May, June and/or July (See S5 Fig). The percentage cover did tend to lower with increasing field margin complexity but again the effect was weak. The ditch component contained more species that flowered in May, June, and/or July than the shrub component and also contained more species that flowered in July, August and/or September than the tree component (Fig 6A). Field margin composition was also associated with the number of flowering species sampled. A higher number of species that flower in October were found in margins containing grassy and ditch components than margins containing grassy, woody and ditch components (Fig 6B). Complexity was weakly associated with species number (Fig 6C).

**Fig 7:**
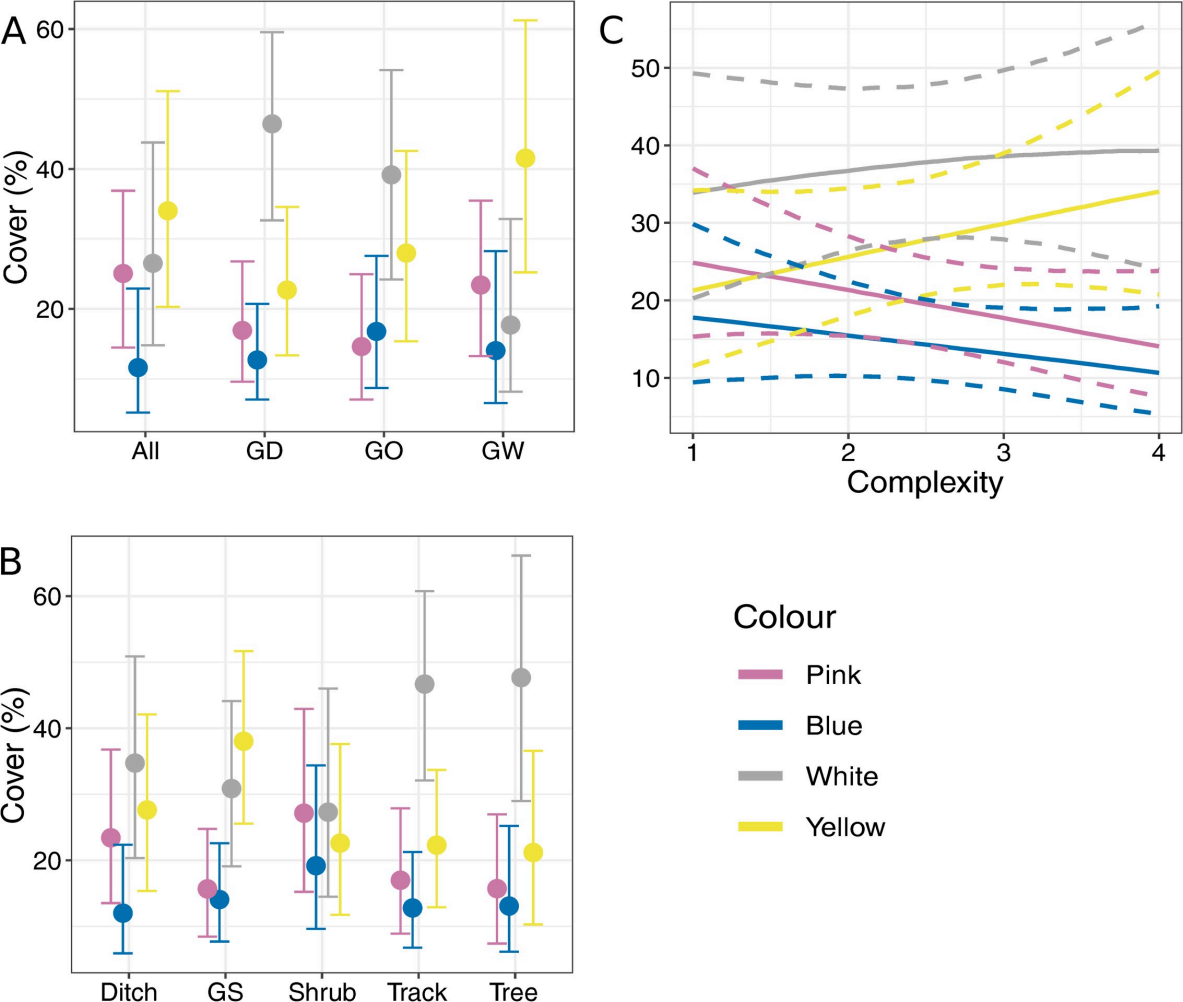
Effect of field margin characteristics on flower colour. Marginal effects of field margin variables on flower colour. Error bars and bands represent 89% credible intervals. A: field margin composition; B: field margin component type; C: field margin complexity. GS: grass strip; All: margin contains grassy, woody and ditch components; GD: margin contains grassy and ditch components; GO: margin contains grassy component(s); GW: margin contains grassy and woody components.

Sampled field margins composed of a ditch and grassy margins had more white flowers than margins composed of trees and grassy margins (Fig 7A). The percentage cover of white flowers differs from that of the blue flowers and pink flowers in margins composed of a ditch and grassy components. There were more white flowers than pink flowers and blue flowers in track and tree components, while grass strips contained more yellow flowers than pink flowers and blue flowers (Fig 7B). More yellow flowers than blue flowers were found in the most complex field margins (Fig 7C).

## Discussion

In this study, we found that the adjacent land-use type had an important effect on the composition of the vegetation found in the herbaceous layer. This factor is linked to management practices of both the fields and the field margins as field margin management differed depending on whether the margin was located near cropped or grazed fields. Management is an important factor in determining the distribution of plant species [24]. Field margins near cropped fields were often mowed and contained fewer woody components, possibly as not to impede the movement of agricultural vehicles from one field to another. On the other hand, field margins located near grazed fields often contained woody components and were less often mowed than those located adjacent to cropped fields. This can be seen in the indicator species analysis where the species that was the most associated with field margins adjacent to grazed fields was *Rubus* sp., which was left to grow in unmowed field margins. The effect of adjacent land-use on vegetation composition cannot be explained by field margin management alone however. Field margins adjacent to cropped fields were less fertile than those adjacent to grazed fields. This could be explained by the presence of animals in the grazed fields. In field margins near the cropped fields, *Trifolium repens* was commonly found and was also often found in the cultivated fields. The abundance of the other indicator species for these field margins, *Equisetum telmateja*, could be explained by the position of the cropped fields at the southern confinement of the study area near the forest. Its preference for humid soil and its higher tolerance to shade than many of the other weeds found, would make these field margins a suitable habitat for this species. The species most strongly associated with field margins adjacent to the road, *Dactylis glomerata*, was also found by Aavik et al. [20] to be common in such field margins. However, no clear type of species linked to field margins adjacent to the road was distinguishable. Indeed, in the ordination, the field margins adjacent to the road are not as clearly grouped together as they are for the margins adjacent to the grazed or cropped fields. These results show that diversifying farming practices and management can lead to differences in vegetation assemblages in surrounding field margins. Although not investigated in this study, it is worth noting that biotic interactions between species can also shape communities and select for functional vegetation types [51].

The main variation in species composition was not explained by any of the variables tested. Nevertheless, we can speculate on possible factors that may explain this variation by observing the distribution of the ordination objects in relation to the first axis. Previously, Aavik and Liira [21] found that landscape structures had a greater effect on species composition than field margin characteristics. Landscape structure was not a factor in our study, however, field margins found on the extreme right of the biplot are nearly all located near the forest, while on the other end of the biplot we find many field margins adjacent to the road. A possible reason for this is that the forest provides shade and humid conditions compared to the dry habitat created by the road [21]. The presence of hygrophytes such as *Equisetum telmateja*, *Carex punctata* and *Mentha suaveolens* on the positive end of the first axis supports this observation. Although the study was conducted at the farm scale to minimize the effect of landscape structures, the influence of landscape structures on vegetation composition seems to have been detected anyway. This hypothesis and the fact that adjacent land-use type was the measured variable that most influenced vegetation composition suggests that field margin vegetation communities in our study are more reactive to landscape factors than local factors. It is possible that in a simpler landscape, shrubs and trees have more influence on vegetation composition.

In our study, we did not find a clear effect of field margin components on vegetation composition apart for the effect of field margin complexity. In the ordination biplot, complex margins were found in the centre while simple ones were found on the top and bottom. This means that simple margins had more extreme soil and management conditions and hosted species characteristic of the specific conditions created in these margins. For example, *Rubus sp*. dominated near grazed margins and *Cynodon dactylon* was characteristic of margins with a lighter sandy soil (Figs 1 and 2). The complex margins hosted species from all margin types, and there were no species characteristic of complex margins. This suggests that complex margins are an import reservoir for the plant diversity in agricultural landscapes with small amounts of semi-natural habitat.

Habitat heterogeneity is important in maintaining biologically diverse agroecosystems and our findings complement this as different types of plants were present in different margin types [52]. More grassweeds were found in track components than in tree components. This would suggest that the disturbance caused by vehicles as well as the reduction in shade favours grassweeds in the study area. Although plant preference for light varied little between vegetation types, the track component did have a tendency to contain more light favouring species than the tree component (S6 and S7 Figs). A similar result was found in Spain where a decrease in woody and evergreen perennials in field margins and intensive agricultural practices resulted in a higher abundance of weeds in margins [53]. Furthermore, we found a strong increase of dicot weeds at the expense of grassweeds along a gradient of field margin complexity in cropped fields. As field margin complexity increases, the likelihood of the field margin containing shrubs, trees or both also increases and, hence, as does the likelihood of the margin having more shade. As expected, field margins were dominated by ruderal species, however, the shrub component had a more balanced species composition in terms of CSR strategies [4]. This might be due to lower luminosity and the presence of dense vegetation such as bramble that may not leave enough space for ruderal species to establish.

In general, effect traits varied little between field margin types. Although we did find that simple field margins composed of only grass strips and or vehicles tracks clearly favoured the presence of hymenoptera flowers *sensu* Müller [32] over other flower types. This result, however, does not take into account the flowers produced by vegetation above the herbaceous layer. For example, *Robinia pseudoacacia* was present in some field margins and is considered to produce hymenoptera flowers. The shrub component produced the most even vegetation composition in terms of flower colour along with the ditch component, however, the ditch component is more resilient in floral resource provisioning than the shrub component from May to July. The track and tree component produced more white flowers than pink or blue flowers. Differences in flower colours in nature are due to an evolutionary adaptation to attract insects [54]. Many syrphids are attracted to white umbellifer flowers and having them in a field margin may increase the potential of attracting these beneficial insects [55]. The commonly occurring syrphids *Episyrphus balteatus* L. and *Eristalis tenax* L. were found to have a preference for yellow flowers, a type of flower which increased in abundance with field margin complexity [56].

The use of functional traits in our study provides an effective way of describing plant assemblages while also providing information on potential ecosystem service provisioning [11, 57]. A possible limitation with employing a functional trait approach is that functional traits can vary within species [25, 58]. In our study, this was not a problem as we did not use traits such as species leaf area or plant height which may vary much more between individuals than, for example, flower colour. The estimation for CSR would be the trait used that had the biggest potential to vary within species, however, the lack of a strong gradient in our study site would suggest that it did not vary much [25]. All the same, although the functional traits used in this study allowed us to detect an effect of field margin properties on vegetation composition, interpretation of the results must be done knowing the limitations of using this method. Furthermore, the effect traits used describe the flowers in terms of flower colour and which type of insect feeds on it but do not give an indication on flower size, flower number per plant, or how much nectar/pollen is produced. This means that having a higher percentage of diptera flowers in one margin than another does not necessarily mean that a higher amount of floral resource is provided for flies by that margin than another. To overcome this hurdle, one possibility could have been to combine the traits with an indicator of nectar and pollen quantity produced by species, however, this would require density data as opposed to percentage cover for the vegetation and specific data on nectar and pollen production which are not readily available. Alternatively, flower sampling could have been conducted, however, this approach focusses only on flowers visited by insects, while our vegetation survey allows a more complete evaluation of potential ecosystem services that can be provided by the field margins.

The information acquired in this study could be used to enhance ecosystem service provisioning in agroecosystems. For example, in our study site, the abundance of grassweeds could be reduced by having less vehicle tracks in the margins. This might not be feasible for all farms as machinery movement is fundamental and cannot easily be changed. Alternatively, increasing the amount of different component types in the field margin can also lead to a reduction in grassweeds. Simple grassy margins can be used to attract hymenopterans such as bees. Using this knowledge can be especially effective in simple landscapes where local factors have more of an influence on insect abundance and species richness [59, 60]. In general, different field margin types offer different potential services and, therefore, a diversity of margins in terms of field margin component type, field margin component composition and field margin complexity should be promoted to obtain a diversity of ecosystem services.

## Conclusions

The main hypothesis of this study was that structural field margin characteristics influenced field margin vegetation composition of the herbaceous layer. In our study area, we found that agricultural management and not so much field margin component type or the composition of the field margin in terms of component type influenced the species composition. However, the influence of management on vegetation community was tempered by the complexity of the field margins in terms of component types. It is also likely that surrounding landscape elements had an effect on vegetation composition.

Field margin characteristics influenced the distribution of both response and effect functional traits. Although field margin components had little influence on species composition, field margin component type, field margin component composition and field margin complexity had an effect on functional trait distribution. This is especially true for response traits with an increase in field margin complexity generally associated with a reduction in grassweeds and stress-tolerant species and an increase in dicotyledon weeds and woody species as well as ruderal species.

Finally, we recommend a diversity of field margin composition should be used as well as a mixture of simple and complex margins. This diversity is especially important in simple landscapes. We also recommend that future studies should be conducted in different landscapes as the landscape type can have an influence on vegetation composition and its functional traits.

## Supporting information

S1 Dataset (ZIP)

S1 Table Spatial variation in soil factors. Mean and standard deviation for each soil factor measured. N: Nitrogen; SOM: Soil organic matter; Capacity: soil water holding capacity.
(DOCX)

S2 Table Field margin component types. Definition of the different field margin components sampled.
(DOCX)

S3 Table Müller’s flower class. Description of Müller’s flower class.
(DOCX)

S4 Table Model descriptions. Description of models retained for analysis. VegTyp: vegetation typology; CSR: Grime’s CSR strategy; FlTyp: flower type; Period: period when vegetation is in flower; Colour: flower colour; Compon: field margin component type; Compos: field margin component composition; Complex: field margin complexity. (1|ALU) indicates that the intercept varied with adjacent land use type as a group effect. (explanatory variable|ALU) indicates that the slope of the explanatory variable varied with adjacent land use type as a group effect.
(DOCX)

S5 Table Species list. List of species found in the study with the percentage of field margins where they were found (frequency) and their mean percentage cover in those field margins.
(DOCX)

S1 Fig Map of the study site. The red polygon represents the study site.
(PNG)

S2 Fig Photograph of a field margin containing the trees and ditch components. (JPG)

S3 Fig Photograph of a field margin containing the vehicle track component. (JPG)

S4 Fig Photograph of a field margin containing the shrubs component. (JPG)

S5 Fig Effect of field margin characteristics on vegetation in flower in May, June, and/or July. Marginal effects of field margin variables on the percentage cover of vegetation that is in flower in May, June, and/or July. Error bars and bands represent 89% credible intervals. A: field margin composition; B: field margin component type; C: field margin complexity in cropped fields; D: field margin complexity in grazed fields. D: ditch; GS: grass strip; S: shrub; TE: tree; TK: track; All: margin contains grassy, woody and ditch components; GD: margin contains grassy and ditch components; GO: margin contains grassy component(s); GW: margin contains grassy and woody components.
(PNG)

S6 Fig Effect of light on vegetation type. Result of model representing the variation in indicator values for light with vegetation type. L = Pignatti indicator value for light.
(PNG)

S7 Fig Variation in community weighted means of indicator values for light. Result of a mixed effect model representing the variation in community weighted means of indicator values for light with component type. L = Pignatti indicator value for light. Adjacent land-use type was included as a random effect.
(PNG)
