# Supplementary material for: Structural field margin characteristics affect the functional traits of herbaceous vegetation
Source: PLoS One. 2020 Sep 17;15(9):e0238916. doi: 10.1371/journal.pone.0238916 (PMC7498012; doi:10.1371/journal.pone.0238916)
Supplement: S1 Table — Mean and standard deviation for each soil factor measured. N: Nitrogen; SOM: Soil organic matter; Capacity: soil water holding capacity. (DOCX) [file pone.0238916.s002.docx]

S1 Table. Mean and standard deviation for each soil factor measured. N: Nitrogen; SOM: Soil organic matter; Capacity: soil water holding capacity.

|  | N (‰) | SOM % | Capacity % | Clay % | Loam % | Sand % |
| --- | --- | --- | --- | --- | --- | --- |
| Ditch | 1.80 ± 0.20 | 2.84 ± 0.31 | 28.76 ± 2.67 | 12.34 ± 1.56 | 36.89 ± 2.29 | 50.78 ± 3.56 |
| Grass strip | 2.04 ± 0.28 | 3.12 ± 0.71 | 24.13 ± 3.92 | 10.41 ± 2.03 | 27.63 ± 4.09 | 61.96 ± 5.81 |
| Shrub | 2.34 ± 0.23 | 3.78 ± 0.41 | 27.65 ± 1.40 | 7.78 ± 0.62 | 30.96 ± 2.86 | 61.26 ± 2.90 |
| Track | 1.92 ± 0.18 | 2.77 ± 0.34 | 26.66 ± 3.73 | 13.35 ± 2.31 | 29.22 ± 4.25 | 57.43 ± 5.83 |
| Tree | 2.80 ± 0.24 | 4.87 ± 0.52 | 31.35 ± 2.32 | 12.11 ± 1.65 | 30.99 ± 2.30 | 56.90 ± 3.66 |
| Cropped | 1.72 ± 0.15 | 2.44 ± 0.34 | 21.91 ± 2.36 | 10.17 ± 0.94 | 25.68 ± 2.68 | 64.15 ± 3.41 |
| Grazed | 2.51 ± 0.16 | 4.25 ± 0.33 | 31.47 ± 1.07 | 10.49 ± 1.36 | 35.28 ± 1.52 | 54.23 ± 2.53 |
| Road | 2.42 ± 0.14 | 3.91 ± 0.31 | 31.82 ± 1.51 | 14.46 ± 2.07 | 34.31 ± 2.31 | 51.23 ± 3.90 |
| All | 2.35 ± 0.17 | 3.79 ± 0.41 | 31.19 ± 1.28 | 12.22 ± 1.54 | 33.18 ± 2.13 | 54.60 ± 3.11 |
| GD | 1.62 ± 0.25 | 2.59 ± 0.59 | 19.55 ± 2.56 | 10.07 ± 1.52 | 24.84 ± 4.21 | 65.09 ± 5.48 |
| GO | 1.83 ± 0.20 | 2.37 ± 0.40 | 26.34 ± 2.95 | 11.89 ± 2.06 | 29.47 ± 5.04 | 58.64 ± 6.76 |
| GW | 2.50 ± 0.17 | 4.17 ± 0.30 | 29.76 ± 2.09 | 10.41 ± 1.50 | 33.97 ± 1.68 | 55.62 ± 2.74 |
| Total | 2.17 ± 0.69 | 3.44 ± 1.48 | 27.62 ± 8.25 | 11.17 ± 5.01 | 31.14 ± 9.34 | 57.69 ± 12.74 |
