# Supplementary material for: Structural field margin characteristics affect the functional traits of herbaceous vegetation
Source: PLoS One. 2020 Sep 17;15(9):e0238916. doi: 10.1371/journal.pone.0238916 (PMC7498012; doi:10.1371/journal.pone.0238916)
Supplement: S2 Table — Definition of the different field margin components sampled. (DOCX) [file pone.0238916.s003.docx]

S2 Table. Definition of the different field margin components sampled.

| Component | Definition |
| --- | --- |
| Track | Herbaceous strip with marks left by wheels of a vehicle.  The strip should be over 1 m in width. |
| Grass strip | A strip of herbaceous plants.  The strip should be over 1 m in width. |
| Shrubs | Woody plants under 4 m in height. Usually does not possess a trunk.  The percentage of ground covered by shrubs should be over 70%. |
| Trees | Woody plants over 4 m tall. Usually have a trunk.  The canopy cover should be over 70%. |
| Ditch | A long narrow excavation dug in the earth (for drainage).  The ditch should be over 1 m in width. |
