# Supplementary material for: Structural field margin characteristics affect the functional traits of herbaceous vegetation
Source: PLoS One. 2020 Sep 17;15(9):e0238916. doi: 10.1371/journal.pone.0238916 (PMC7498012; doi:10.1371/journal.pone.0238916)
Supplement: S3 Table — Description of Müller’s flower class. (DOCX) [file pone.0238916.s004.docx]

S3 Table. Description of Müller’s flower class.

| Flower type | Abbreviation | Description |
| --- | --- | --- |
| Open nectar flower | Open nectar flw | Flowers with easily accessible nectar. |
| Hidden nectar flower | Hidden nectar flw | Flowers with nectar not easily accessible. |
| Diptera flowers | Diptera flw | Flowers that are typically pollinated by flies. |
| Lepidoptera flowers | Lepidoptera flw | Flowers that are typically pollinated by species of lepidoptera as well as some long-tongued bees and syrphids. |
| Hymenoptera flowers | Hymenoptera flw | Flowers that are typically pollinated by species of hymenoptera. |
| Pollen flowers | Pollen flw | Flowers that produce pollen  that are consumed by insects. |
