# Supplementary material for: Structural field margin characteristics affect the functional traits of herbaceous vegetation
Source: PLoS One. 2020 Sep 17;15(9):e0238916. doi: 10.1371/journal.pone.0238916 (PMC7498012; doi:10.1371/journal.pone.0238916)
Supplement: S4 Table — Description of models retained for analysis. VegTyp: vegetation typology; CSR: Grime’s CSR strategy; FlTyp: flower type; Period: period when vegetation is in flower; Colour: flower colour; Compon: field margin component type; Compos: field margin component composition; Complex: field margin complexity. (1|ALU) indicates that the intercept varied with adjacent land use type as a group effect. (explanatory variable|ALU) indicates that the slope of the explanatory variable varied with adjacent land use type as a group effect. (DOCX) [file pone.0238916.s005.docx]

S4 Table. Description of models retained for analysis. VegTyp: vegetation typology; CSR: Grime’s CSR strategy; FlTyp: flower type; Period : period when vegetation is in flower; Colour: flower colour; Compon: field margin component type; Compos: field margin component composition; Complex: field margin complexity. (1|ALU) indicates that the intercept varried with adjacent land use type as a group effect. (explanotory variable|ALU) indicates that the slope of the explanatory variable varried with adjacent land use type as a group effect.

| Response variable | Explanatory variables | Response distribution |
| --- | --- | --- |
| VegTyp | Compon + (1\|ALU) | Dirichlet |
| VegTyp | Compos + (1\|ALU) | Dirichlet |
| VegTyp | Complex + (Complex\|ALU) | Dirichlet |
| CSR | Compon + (1\|ALU) | Dirichlet |
| CSR | Compos + (1\|ALU) | Dirichlet |
| CSR | Complex + (Complex\|ALU) | Dirichlet |
| FlTyp | Compon + (1\|ALU) | Dirichlet |
| FlTyp | Compos + (1\|ALU) | Dirichlet |
| FlTyp | Complex + (Complex\|ALU) | Dirichlet |
| Period | Compon + (1\|ALU) | Beta |
| Period | Compos + (1\|ALU) | Beta |
| Period  Species richness  Species richness  Species richness | Complex + (1\|ALU)  Compon + (1\|ALU)  Compos + (1\|ALU)  Complex + (1\|ALU) | Beta  Poisson  Poisson  Poisson |
| Colour | Compon + (1\|ALU) | Dirichlet |
| Colour | Compos + (1\|ALU) | Dirichlet |
| Colour | Complex + (1\|ALU) | Dirchlet |
