# Supplementary material for: Structural field margin characteristics affect the functional traits of herbaceous vegetation
Source: PLoS One. 2020 Sep 17;15(9):e0238916. doi: 10.1371/journal.pone.0238916 (PMC7498012; doi:10.1371/journal.pone.0238916)
Supplement: S5 Table — List of species found in the study with the percentage of field margins where they were found (frequency) and their mean percentage cover in those field margins. (DOCX) [file pone.0238916.s006.docx]

S5 Table. List of species found in the study with the percentage of field margins where they were found (frequency) and their mean percentage cover in those field margins.

| Species name | Frequency (%) | Mean (%) |
| --- | --- | --- |
| *Agrimonia eupatoria* | 7.5 | 4 |
| *Agropyron repens* | 10 | 6.25 |
| *Apera spica-venti* | 5 | 15.5 |
| *Aristolochia clematitis* | 2.5 | 9 |
| *Artemisia verlotiorum* | 20 | 27.12 |
| *Avena fatua* | 12.5 | 3.75 |
| *Bellis perennis* | 2.5 | 3 |
| *Brachypodium pinnatum* | 15 | 23.25 |
| *Brachypodium sylvaticum* | 32.5 | 11.69 |
| *Bromus tectorum* | 2.5 | 5 |
| *Calamagrostis epigejos* | 2.5 | 1 |
| *Calamintha nepeta* | 5 | 16.5 |
| *Calystegia sepium* | 2.5 | 5 |
| *Carex divulsa* | 10 | 2.5 |
| *Carex flacca* | 7.5 | 3.67 |
| *Carex punctata* | 2.5 | 2 |
| *Cichorium intybus* | 7.5 | 3.33 |
| *Cirsium arvensis* | 7.5 | 3.33 |
| *Cirsium vulgare* | 5 | 3.5 |
| *Clematis vitalba* | 5 | 3 |
| *Coleostephus myconis* | 10 | 14.33 |
| *Convolvulus arvensis* | 5 | 2 |
| *Conyza canadensis* | 5 | 2 |
| *Cornus sanguinea* | 2.5 | 3 |
| *Cynodon dactylon* | 35 | 11.46 |
| *Dactylis glomerate* | 35 | 3.69 |
| *Dipsacus fullonum* | 2.5 | 5 |
| *Dittrichia viscosa* | 10 | 1 |
| *Equisetum telmateia* | 57.5 | 15 |
| *Festuca rubra* | 5 | 4 |
| *Galega officinalis* | 7.5 | 5 |
| *Galium album* | 25 | 3.62 |
| *Galium verum* | 5 | 3 |
| *Gaudinia fragilis* | 22.5 | 5.67 |
| *Hedera helix* | 7.5 | 7.67 |
| *Helminthotheca echioides* | 25 | 3.11 |
| *Holcus lanatus* | 20 | 6.25 |
| *Hordeum murinum* | 5 | 3 |
| *Hordeum secalinum* | 2.5 | 5 |
| *Imperata cylindrica* | 2.5 | 49 |
| *Iris pseudacorus* | 5 | 3.5 |
| *Juncus depauperatus* | 2.5 | 3 |
| *Lathyrus hirsutus* | 2.5 | 3 |
| *Leucanthemum vulgare* | 17.5 | 6.42 |
| *Lolium multiflorum* | 10 | 5.75 |
| *Lolium perenne* | 7.5 | 3 |
| *Lolium rigidum* | 5 | 8.5 |
| *Lotus corniculatus* | 2.5 | 3 |
| *Lotus uliginosus* | 2.5 | 1 |
| *Lythrum salicaria* | 2.5 | 3 |
| *Medicago lupulina* | 10 | 2.25 |
| *Medicago sativa* | 5 | 3 |
| *Melissa officinalis* | 7.5 | 2.67 |
| *Mentha spicata* | 7.5 | 9 |
| *Mentha suaveolens* | 5 | 3.5 |
| *Paspalum paspaloides* | 2.5 | 3 |
| *Petasites albus* | 2.5 | 3 |
| *Phleum pratense* | 20 | 10 |
| *Phragmites australis* | 2.5 | 5 |
| *Plantago lanceolata* | 15 | 3 |
| *Plantago major* | 7.5 | 2 |
| *Poa pratensis* | 7.5 | 7 |
| *Potentilla reptans* | 37.5 | 7.07 |
| *Prunella vulgaris* | 12.5 | 4.33 |
| *Pulicaria dysenterica* | 22.5 | 5.25 |
| *Rubus* sp. | 47.5 | 6.67 |
| *Rumex acetosa* | 2.5 | 2 |
| *Sherardia arvensis* | 2.5 | 2 |
| *Sorghum halepense* | 7.5 | 3 |
| *Stipa trichotoma* | 7.5 | 1.67 |
| *Teucrium scorolium* | 10 | 3.25 |
| *Torilis japonica* | 22.5 | 3.11 |
| *Trifolium badium* | 10 | 3 |
| *Trifolium fragiferum* | 2.5 | 2 |
| *Trifolium repens* | 47.5 | 5.5 |
| *Ulmus minor* | 2.5 | 5 |
| *Valeriana officinalis* | 5 | 3 |
