# Supplementary figures and images for: Structural field margin characteristics affect the functional traits of herbaceous vegetation
Source: PLoS One. 2020 Sep 17;15(9):e0238916. doi: 10.1371/journal.pone.0238916 (PMC7498012; doi:10.1371/journal.pone.0238916)

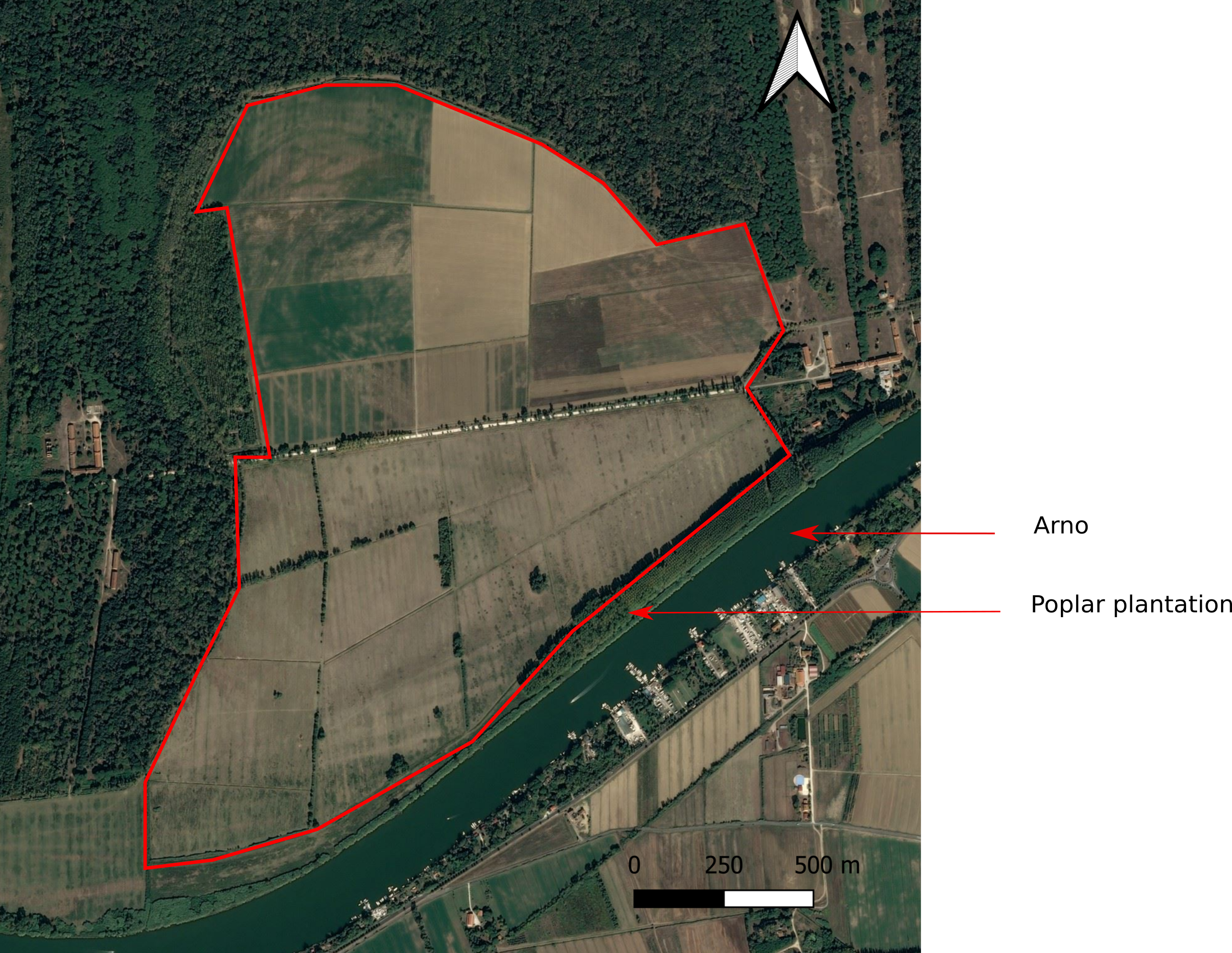

Supplement: S1 Fig — The red polygon represents the study site. (PNG) [file pone.0238916.s007.png]

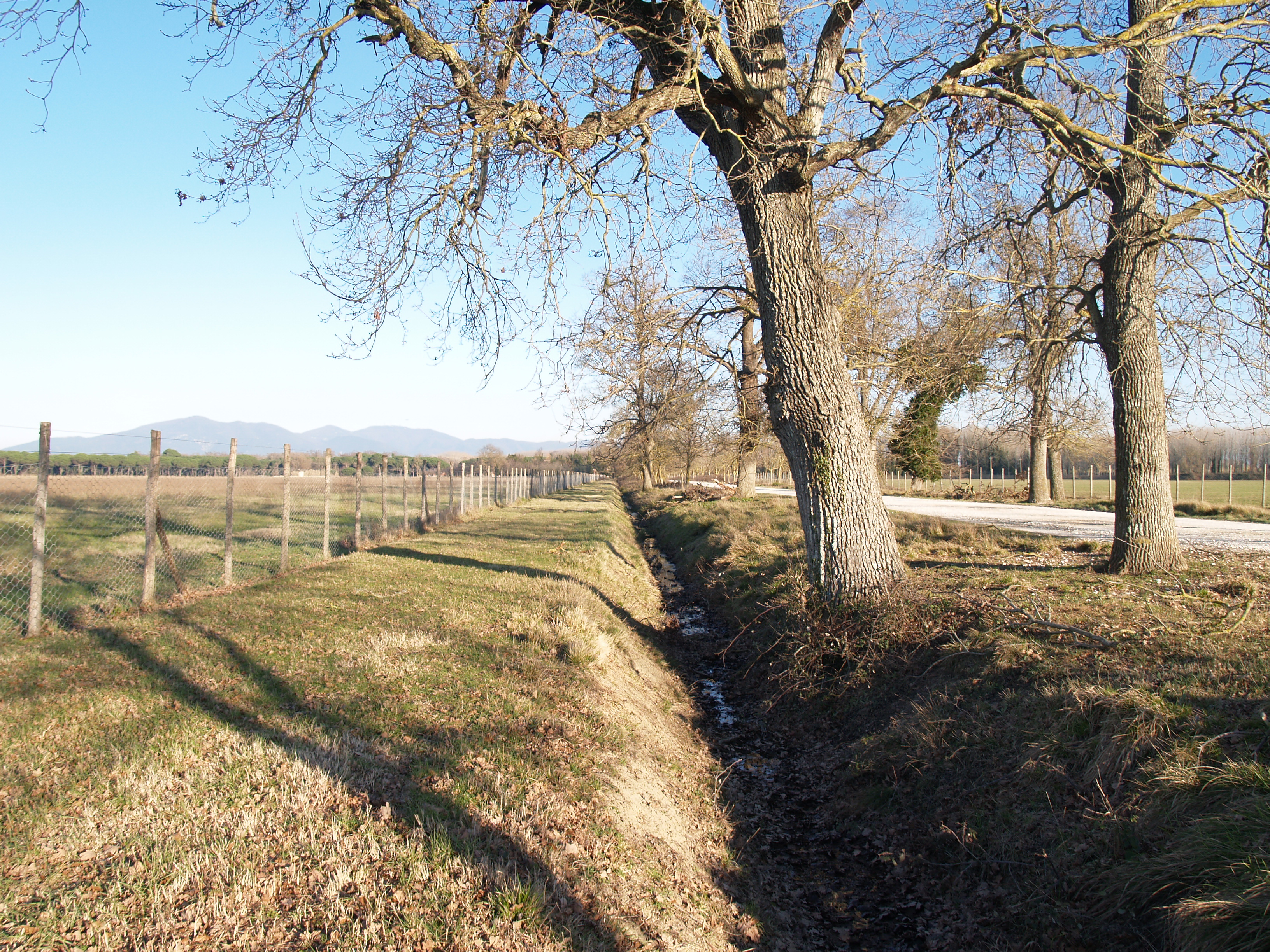

Supplement: S2 Fig — (JPG) [file pone.0238916.s008.JPG]

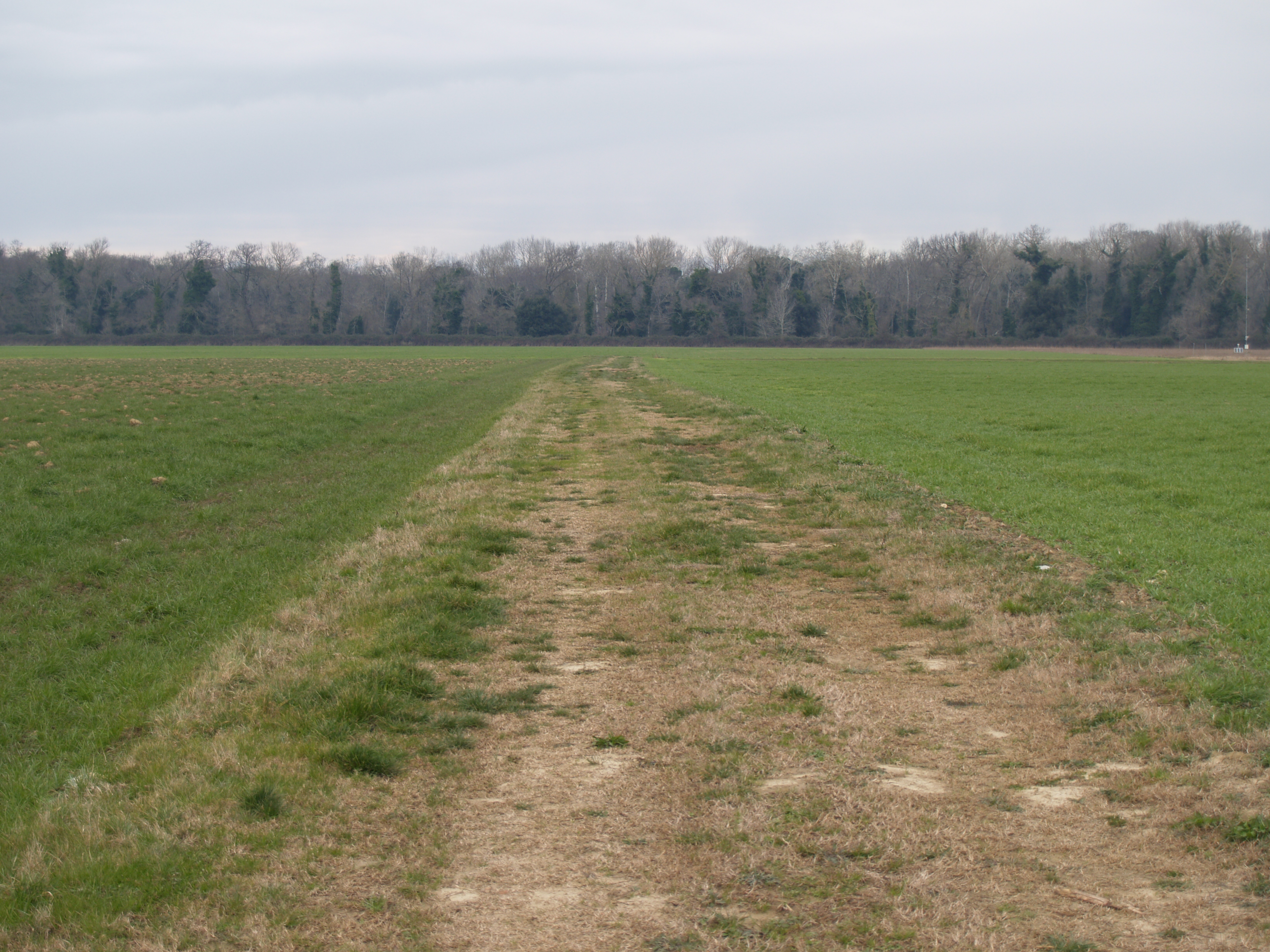

Supplement: S3 Fig — (JPG) [file pone.0238916.s009.JPG]

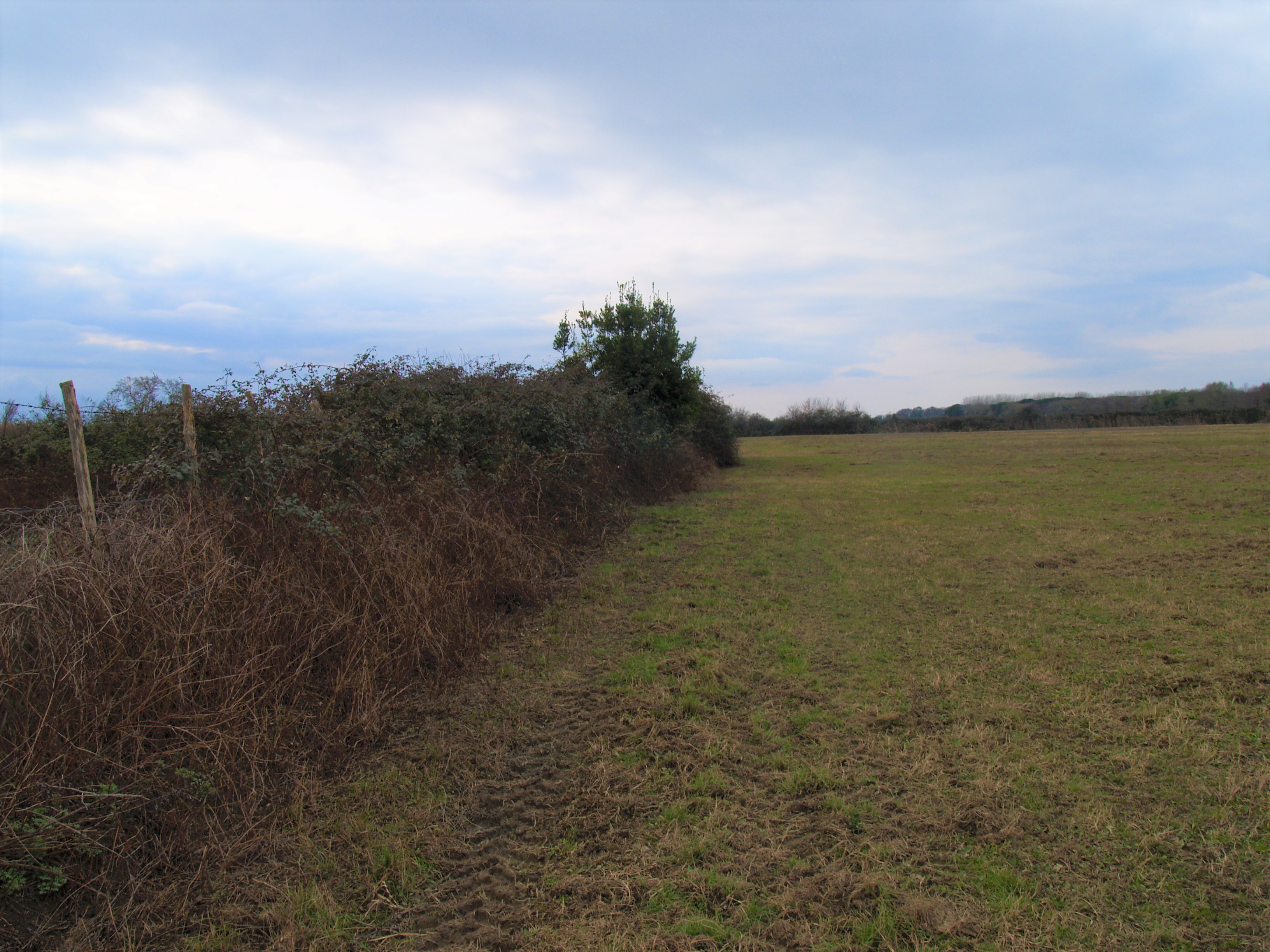

Supplement: S4 Fig — (JPG) [file pone.0238916.s010.jpg]

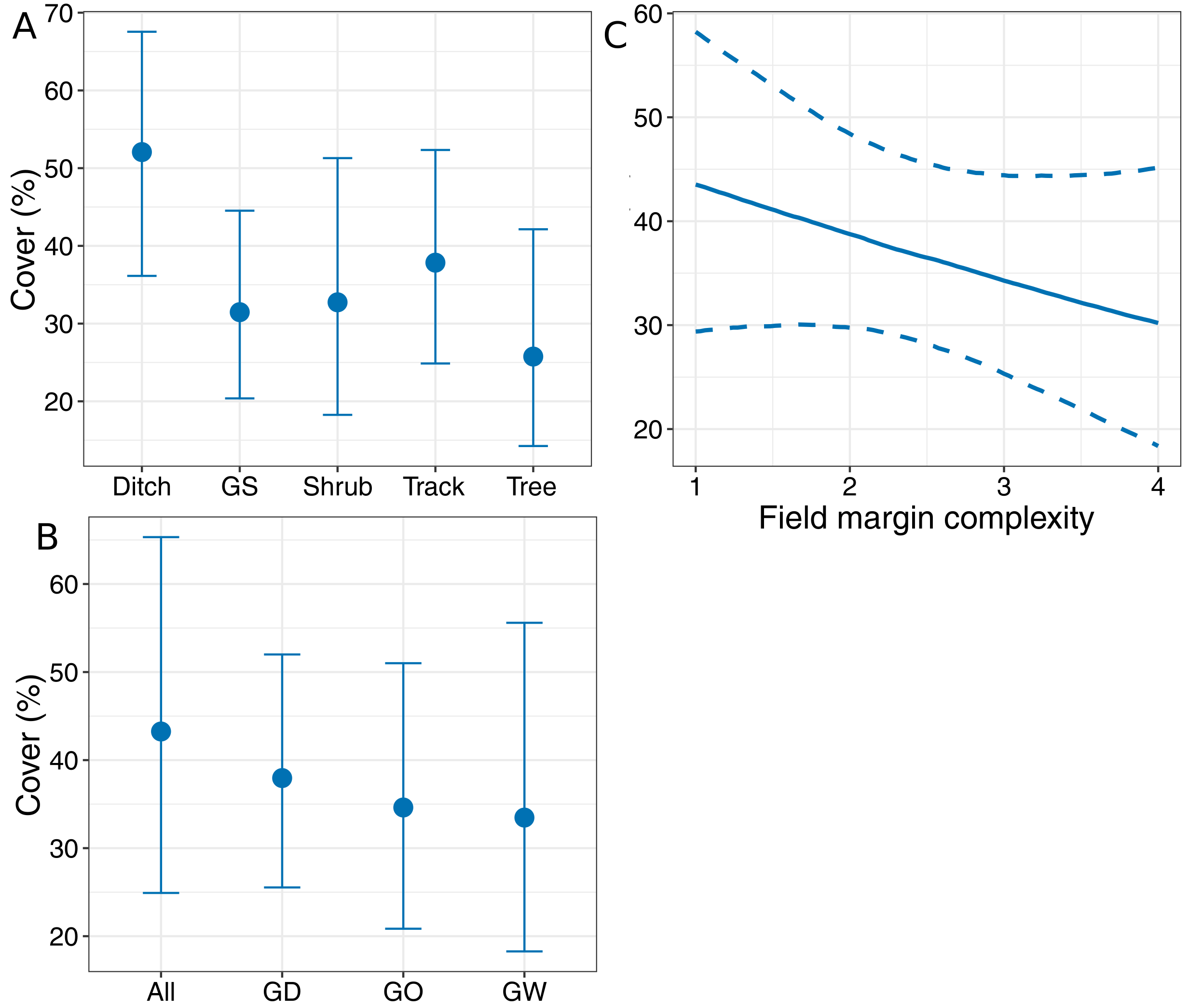

Supplement: S5 Fig — Marginal effects of field margin variables on the percentage cover of vegetation that is in flower in May, June, and/or July. Error bars and bands represent 89% credible intervals. A: field margin composition; B: field margin component type; C: field margin complexity in cropped fields; D: field margin complexity in grazed fields. D: ditch; GS: grass strip; S: shrub; TE: tree; TK: track; All: margin contains grassy, woody and ditch components; GD: margin contains grassy and ditch components; GO: margin contains grassy component(s); GW: margin contains grassy and woody components. (PNG) [file pone.0238916.s011.png]

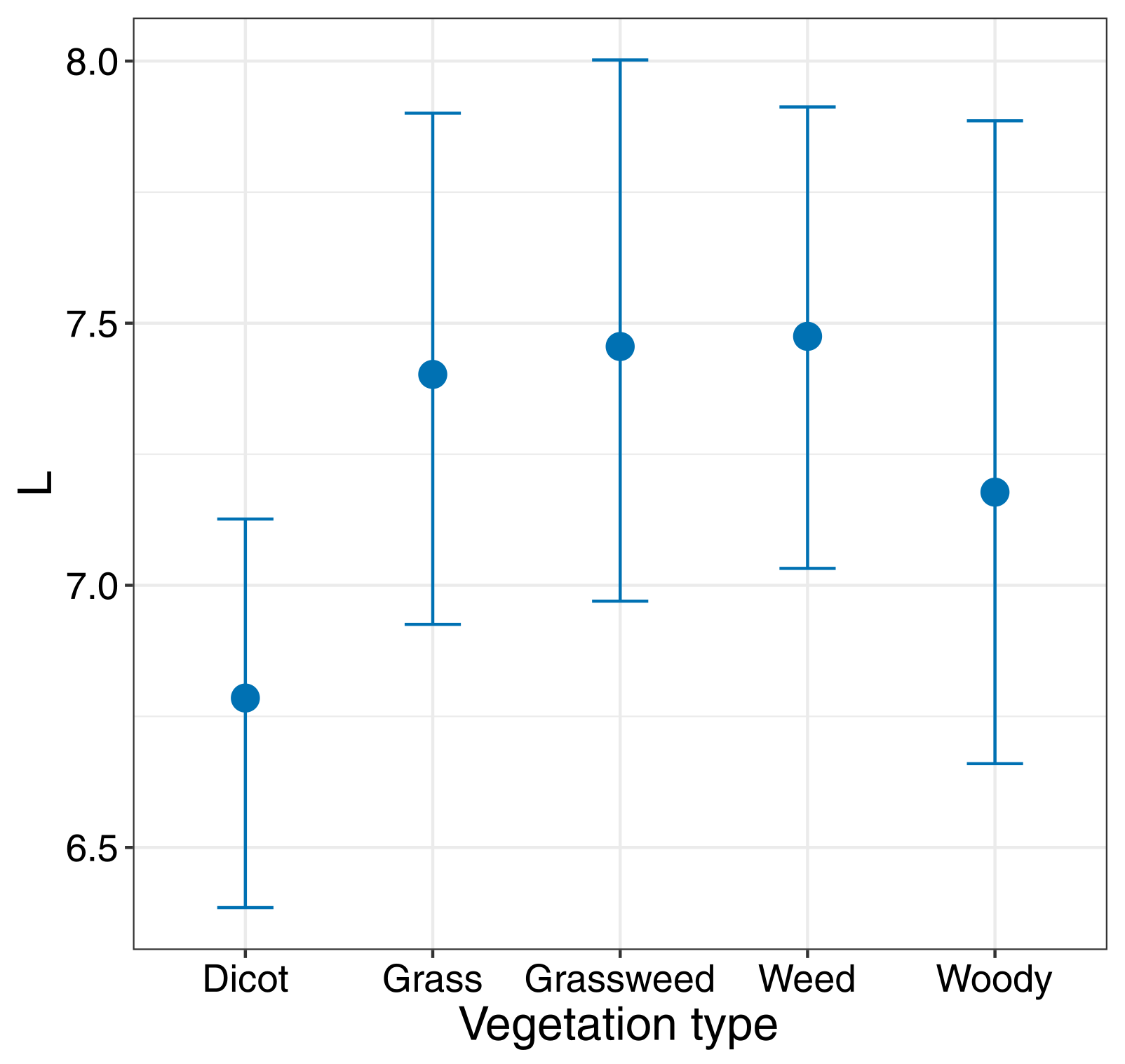

Supplement: S6 Fig — Result of model representing the variation in indicator values for light with vegetation type. L = Pignatti indicator value for light. (PNG) [file pone.0238916.s012.png]

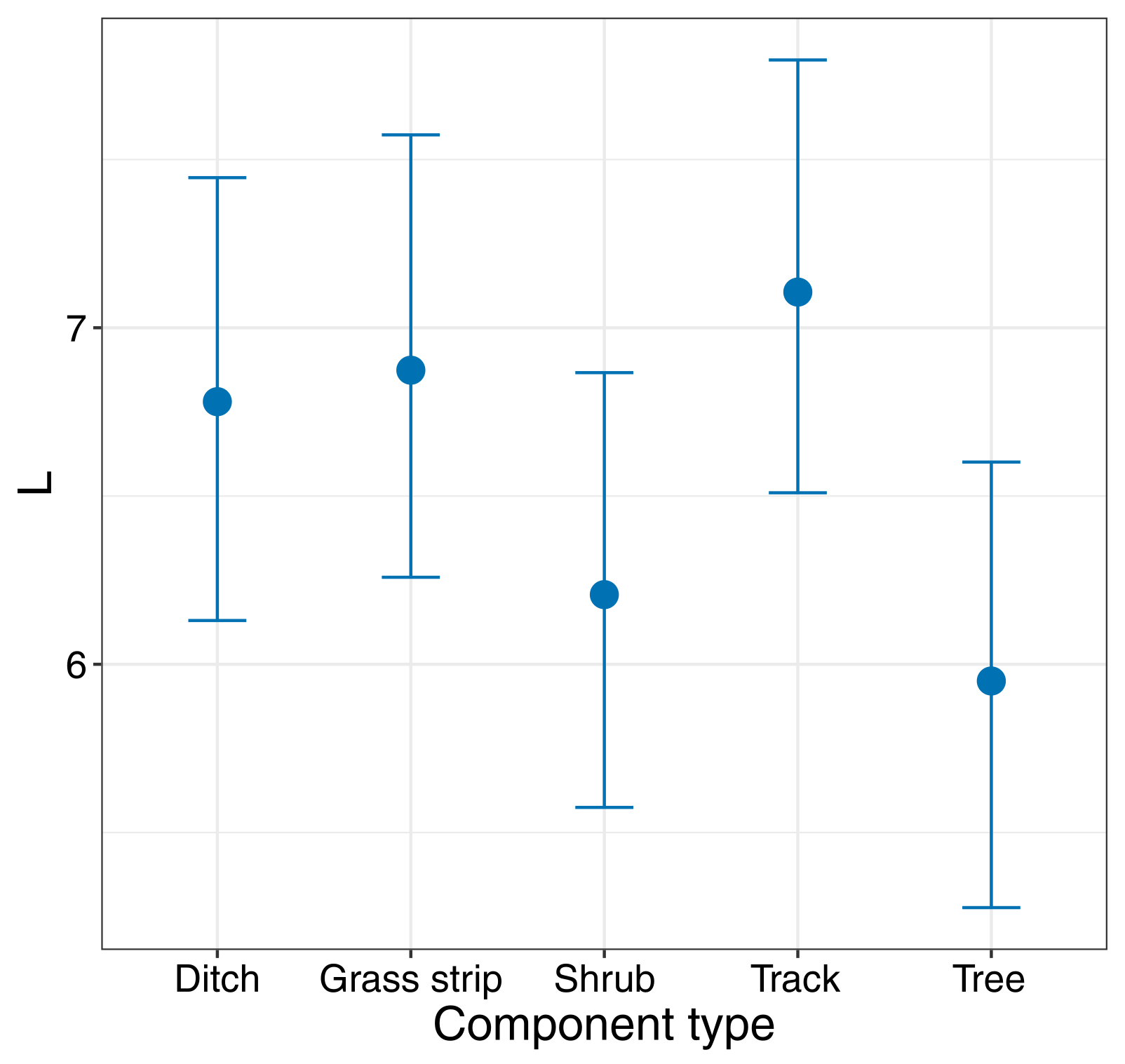

Supplement: S7 Fig — Result of a mixed effect model representing the variation in community weighted means of indicator values for light with component type. L = Pignatti indicator value for light. Adjacent land-use type was included as a random effect. (PNG) [file pone.0238916.s013.png]
